# QSAR studies on PIM1 and PIM2 inhibitors using statistical methods: a rustic strategy to screen for 5-(1H-indol-5-yl)-1,3,4-thiadiazol analogues and predict their PIM inhibitory activity

**DOI:** 10.1186/s13065-017-0269-1

**Published:** 2017-05-19

**Authors:** Adnane Aouidate, Adib Ghaleb, Mounir Ghamali, Samir Chtita, M’barek Choukrad, Abdelouahid Sbai, Mohammed Bouachrine, Tahar Lakhlifi

**Affiliations:** MCNSL, School of Sciences, University Moulay Ismail, Meknes, Morocco

**Keywords:** PIM1, PIM2, 5-(1H-indol-5-yl)-1,3,4-thiadiazol-2-amines, QSAR model

## Abstract

**Background:**

Quantitative structure activity relationship was carried out to study a series of PIM1 and PIM2 inhibitors. The present study was performed on twenty-five substituted 5-(1H-indol-5-yl)-1,3,4-thiadiazols as PIM1 and PIM2 inhibitors having pIC_50_ ranging from 5.55 to 9 µM and from 4.66 to 8.22 µM, respectively, using genetic function algorithm for variable selection and multiple linear regression analysis (MLR) to establish unambiguous and simple QSAR models based on topological molecular descriptors.

**Results:**

Results showed that the MLR predict activity in a satisfactory manner for both activities. Consequently, the aim of the current study is twofold, first, a simple linear QSAR model was developed, which could be easily handled by chemist to screen chemical databases, or design for new potent PIM1 and PIM2 inhibitors. Second, the outcomes extracted from the current study were exploited to predict the PIM inhibitory activity of some studied compound analogues.

**Conclusions:**

The goal of this study is to develop easy and convenient QSAR model could be handled by everyone to screen chemical databases or to design newly PIM1 and PIM2 inhibitors derived from 5-(1H-indol-5-yl)-1,3,4-thiadiazol.

**Graphical abstract:**
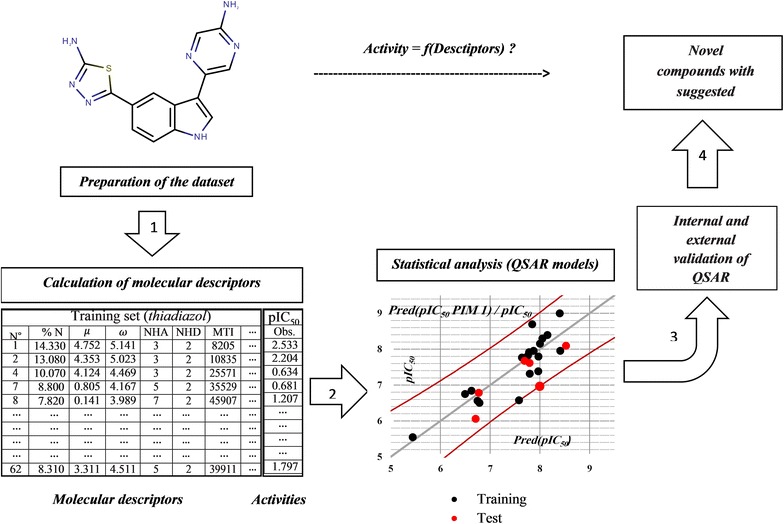
Flow chart of the methodology used in this work.

## Background

PIM1, PIM2 and PIM3 (proviral integration site for moloney murine leukaemia virus) kinases form a three-member subgroup of serine/threonine kinases family, which share a high level of sequence homology and exhibit some functional redundancy. They attracted recent attention for their potential role in tumorigenesis, tumor cell survival and resistance to antitumor agents, thus, these findings make them an attractive target for cancer therapy [1, 2].

In the literature, several classes of molecules as pyrazines [3], cinnamic acid [4] and pyrrolo carbazole [5] have been designed and synthesized to be able to inhibit the PIM1 and PIM2 as well as to exhibit an anticancer activity, and they have been studied with different approaches so far, but this way is regarded as time consuming and very costly. Hence, in order to reduce time and cost also, to design more potent PIM inhibitors, theoretical research can circumvent these difficulties and allow obtaining precise data while taking advantage of the rapid progress of computing chemical descriptors, which can be obtained easily from publicly available software and servers. Therefore, developing predictive quantitative structure activity relationship (QSAR) models to predict the activity of new synthesized or designed PIM inhibitors is highly desired.

In this context, the QSAR of thiadiazoles still receives considerable attention because these agents represent a large family of multi-biological activity substances and continue to be a source of new drugs as witnessed over recent decades. Thus, it is important to extend these findings with all available data. Recently, a series of some potent PIM1 and PIM2 inhibitors have been designed and reported by Bin Wu and al. [6]. To the best of our knowledge, no QSAR studies have been carried out based on the reported activities of this series. That prompted us to aim an in silico study based on it, as well as to generalize beyond the data to screen and predict inhibitory activity of other analogues molecules.

Quantitative structure–activity relationship (QSAR) has been widely used last years in drug discovery and drug design by medicinal chemists [7, 8] and in various practical applications [9, 10] to provide quantitative analysis of structure and biological activity relationships of compounds. Different QSAR studies were reported to identify important structural features responsible for the biological activity and to develop predictive models for diverse chemicals by different authors [11, 12]. Thus, it becomes necessary to develop a QSAR model for the prediction of activity before synthesis of new PIM1 and PIM2 inhibitors. Because, a successful QSAR model is not only helps to understand relationships between the physicochemical properties and biological activity of any class of molecules, but also provides researchers a deep analysis about the lead molecules to be used in further studies [13].

Therefore, the current research aims to derive highly correlation models, which explain the relationship between the anticancer activity, and the structure of twenty-five compounds based on physicochemical descriptors using several chemometric methods such as genetic function algorithm GFA, multiple linear regression MLR. Consequently, the principal goal of this work is to develop easy and convenient QSAR model could be handled by everyone for screening or designing newly PIM1 and PIM2 inhibitors derived from thiadiazoles.

## Methods

**Fig. 1:**
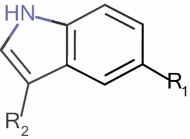
The chemical structure of the studied compounds

PIM1 and PIM2 inhibitory activities of a series of twenty-five of 5-(1H-indol-5-yl)-1,3,4-thiadiazol-2-amine derivatives were taken from literature [6] each activity was expressed as IC_50_ (µM) then was converted to pIC_50_ as pIC_50_ = −log IC_50_. Figure 1 and Table 1 show the substituted structures of the studied compounds. For modeling purpose, the data set was split into two sets. Nineteen molecules were randomly chosen to build the quantitative model (training set), and the remaining molecules were used to test the performance of the established model (test set) for both activities. Additionally leave-one-out protocol and Y-randomization were carried out to study the stability of the chosen training sets.

**Table 1:**
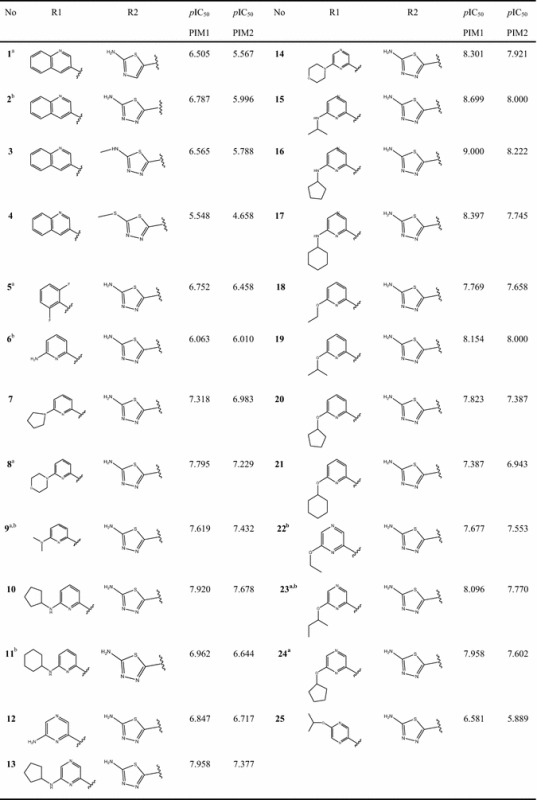
Chemical structures and anti-cancer activities of substituted 5-(1H-indol-5-yl)-1,3,4-thiadiazol-2-amine derivatives

^a, b^Are the test sets for PIM1 and PIM2 inhibitory activities respectively

### Molecular descriptors

All modeling studies were performed using the SYBYL-X 2.0 molecular modeling package (Tripos Inc., St. Louis, USA) running on a windows 7, 32 bits workstation. Three-dimensional structures were built using the SKETCH option in SYBYL. All compounds were minimized under the Tripos standard force field [14] with Gasteiger-Hückel atomic partial charges [15] by the Powell method with a convergence criterion of 0.01 kcal/mol Å. To describe the compound structural diversity and in order to obtain validated QSAR models, the optimized structures were saved in sdf format and transferred to PaDEL server [16] to calculate topological descriptors encode the chemical properties of each compound. Among the calculated descriptors only three descriptors have been chosen as relevant to describe each studied inhibitory activity (Table 2).

**Table 2:** The three relevant molecular descriptors used in each best QSAR model for each activity

Selected descriptors for PIM1 inhibitory activity		Selected descriptors for PIM2 inhibitory activity	
AATS0p	Autocorrelation	GATS8v	Geary autocorrelation of lag 8 weighted by van der Waals volume
maxHBint8	Atom type electrotopological state	AATS3i	Autocorrelation
GATS8v	Geary autocorrelation of lag 8 weighted by van der Waals volume	VR1_Dzm	Barysz matrix

### Methodology

After the calculation of all descriptors from PaDEL server, a genetic function algorithm (GFA) analysis for variable selection was applied on the molecular descriptors’ set to choose only the appropriate ones to describe each activity [17]. Subsequently, the number was reduced to three, which is reasonable considering the number of molecules used to build the models according to the rule of five [18]. Then, those three chosen descriptors were used as input to perform an MLR study on each activity until a valid model including: the critical probability *p* value <0.05 for all descriptors and for the complete model, the Fisher criterion, the determination coefficient, the mean squared error, the multi-colinearity test, and the internal, external validations, in addition to the Y-randomization. Later, those descriptors were also exploited to generate the applicability domain to describe the chemical space for each model.

### Statistical analysis

In the present study XLSTAT version 2013 [19] was used to perform multiple linear regression (MLR), which is a statistical method aimed to establish a mathematical relationship between a property of a given system and a set of molecular descriptors that encode chemical information. A genetic function algorithm tool was used for variables selection [17], which is a mathematical technique served to reduce the number of variables used in the data set, as well as to select only the pertinent ones, in which mutation probability was 0.5 the smoothing parameter was 1.0, and cross over probability was 1.0. GFA in this study serves to select significant molecular descriptors from vast number of variables.

### Validation

The main objective of a QSAR study is to obtain a model with the highest predictive and generalization abilities. Therefore, two principals (internal validation and external validation) were carried out in order to evaluate the predictive power of the developed QSAR models. For the internal validation, the leave-one-out cross-validation process (Q^2^) was used to evaluate the stability and the internal capability of the proposed models in the present study. A high Q^2^ value means a high internal predictive power of a QSAR model and a good robustness. Nevertheless, the study of Globarikh [20] indicated that there is no correlation between the value of Q^2^ for the training set and predictive ability of the test set, revealing that the Q^2^ is still inadequate for a reliable estimate of model predictive power for all new chemicals. Thus, the external validation regards the only way to determine both the generalizability and the true predictive power of QSAR models for new chemicals. For this reason, the statistical external validation process was applied to the developed models using a test set as described by Globarikh and Tropsha; Roy and Roy [20–22].

### Y-randomization test

The obtained models were further validated by the Y-Randomization method [23]. In which the dependent vector (pIC_50_) is randomly shuffled many times and after every iteration, a new QSAR model is developed. The new QSAR models are expected to have lower Q^2^ and R^2^ values than those of the original models. This technique is carried out to eliminate the possibility of the chance correlation. If higher values of the Q^2^ and R^2^ are obtained, it means that an acceptable QSAR cannot be generated for this data set because of the structural redundancy and chance correlation.

## Results and discussion

### Data set for analysis

A QSAR study was carried out for the first time on twenty-five of 5-(1H-indol-5-yl)-1,3,4-thiadiazol-2-amine derivatives, in order to establish quantitative relationships between their structures and their PIM1 and PIM2 inhibitory activities. The three selected descriptors for each model are shown in Table 2.

### Multiple linear regressions MLR

Based on the selected molecular descriptors two mathematical linear models were proposed to predict quantitatively the physicochemical effects of substituents on the PIM1 and PIM2 inhibitory activities using linear regression. In total, nineteen molecules were placed in the training set to build the QSAR models, and the six molecules composed the test set,

$$Y = a_{0} + \sum _{i = 1}^{n} a_{i} x_{i} .$$

For the PIM1 inhibitory activity the best linear model contains three molecular descriptors: GATS8v, AATS0p and maxHBint8 and it is represented by the following equation:

1 $$pIC50 = 6.92 - 5.84 \times \left( {AATS0p} \right) - 0.27 \times \left( {maxHBint8} \right) + 1072 \times (GATS8v)\varvec{ } \varvec{ }$$

N = 19, R = 0.87, R^2^ = 0.726, Q^2^ = 0.60, MSE = 0.221, F = 16.04, P < 0.0001.

For the PIM2 inhibitory activity the best linear model contains three molecular descriptors: GATS8v, AATS3i and VR1_Dzm and it is represented by the following equation:

2 $$pIC50 = - 32.31 + 12.8 \times (GATS8v) + 0.16 \times (AATS3i)\varvec{ } - 8.48 \times (VR1\_Dzm)\varvec{ } \varvec{ }$$

N = 19, R = 0.91, R^2^ = 0.825, Q^2^ = 0.73, MSE = 0.184, F = 23.85, P < 0.0001.

R^2^ is the coefficient of determination, F is the Fisher statistic and MSE is the mean squared error. Higher coefficient of determination and lower mean squared error indicate that the model is more reliable. A P smaller than 0.05 means that the obtained equation is statistically significant at the 95% level. The obtained model were cross-validated by their applicable Q^2^ values (Q^2^ = 0.60 and 0.73) respectively, using the leave-one-out (LOO) method. A value of Q^2^ greater than 0.5 is the basic criteria to qualify a model as valid [20].

The multi-collinearity between the above three descriptors for each model was detected by calculating their variation inflation factors VIF as shown in Table 3. Accordingly, it has been found that the descriptors used in the proposed models have very low-inter-correlation. The VIF [24] was defined as 1/(1−R^2^), where R is the coefficient of correlation between one descriptor and all the other descriptors in the proposed model. A VIF value greater than 5.0 indicates that the model is unstable; a value between 1.0 and 4.0 indicates that the model is acceptable.

**Table 3:** Multi-colinearity test

Variables	PIM1 inhibitory activity			PIM2 inhibitory activity		
	AATS0p	maxHBint8	GATS8v	GATS8v	AATS3i	VR1_Dzm
VIF	2.376	2.343	2.081	1.810	1.960	1.168

**Fig. 2:**
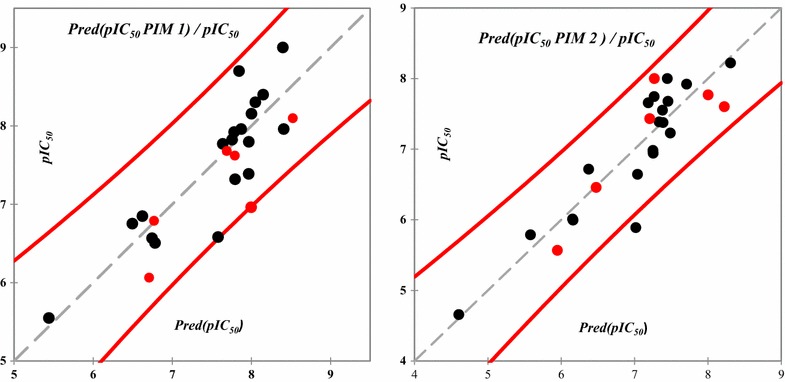
Correlations of observed and predicted activities (training set in *black* and test set in *red*) values calculated using MLR models

The correlations of the predicted and observed activities are illustrated in Fig. 2. The descriptors proposed in Eqs. (1) and (2) by MLR are then used as the input parameters to generate the applicability domains (AD) for both models.

### Applicability domain

The utility of a QSAR model is its accurate prediction ability for new chemical compounds. So, once the QSAR model is built, its domain of applicability (AD) must be defined. A model is regarded valid only within its training domain and only the prediction for new compounds falling within its applicability domain can be considered reliable and not model extrapolations. The most common method to define the AD, it is based on the determination of the leverage value of each compound [22]. The Williams plot [the plot of standardized residuals versus leverage values (*h*)] is used in the present study to visualize the AD of the QSAR model.

$$h_{i} = x_{i}^{T} (X^{T} X)^{ - 1} x_{i}$$

where the x_i_ is the descriptor vector of the considered compound, X is the descriptor matrix derived from the training set descriptor values, the threshold is defined as:

$$h^{*} = \frac{3(k + 1)}{n}$$

where n is the number of compound in the training set, k is the number of the descriptors in the proposed model, a leverage (*h*) greater than the threshold (*h* *) indicates that the predicted response is an extrapolation of the model and, consequently, it can be unreliable.

**Fig. 3:**
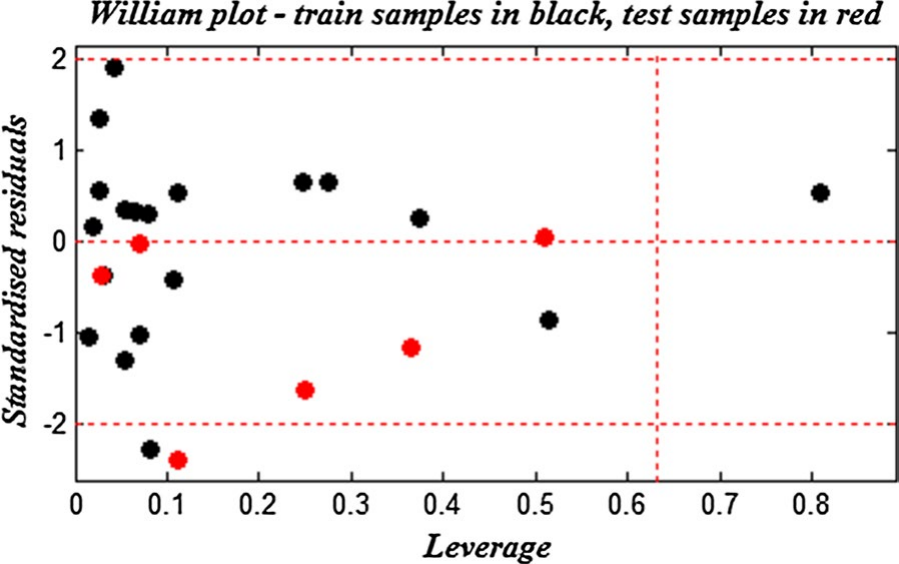
Williams plot for the training set and external validation for the PIM1 inhibitory activity of compounds, listed in Table 1 (*h* * = 0.63 and residual limits ±2)

**Fig. 4:**
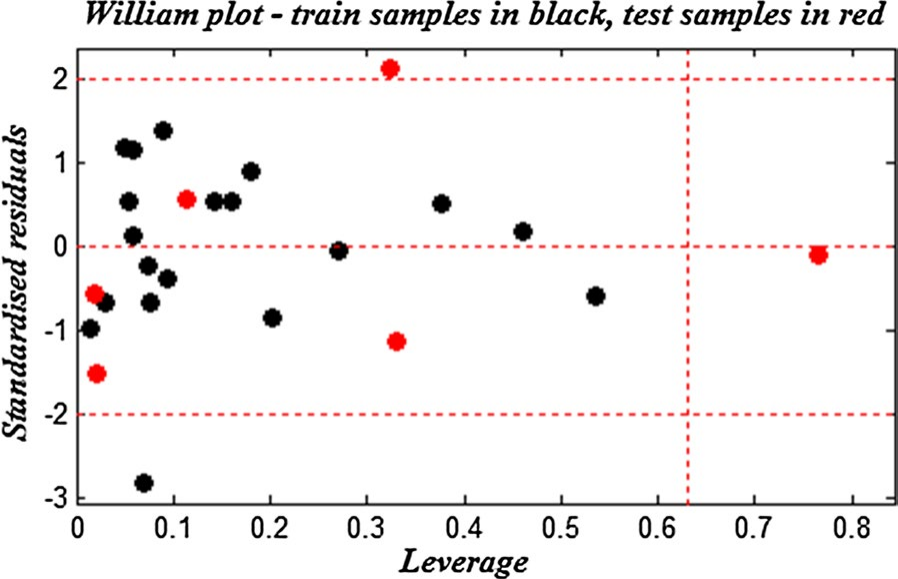
Williams plot for the training set and external validation for the PIM2 inhibitory activity of compounds, listed in Table 1 (h* = 0.63 and residual limits ±2)

The Williams plots of the presented MLR models are shown in Figs. 3 and 4, the applicability domains are established inside a squared area within ±2 standard deviation and a leverage threshold *h* * of 0.63 for both models.

As shown in the developed Williams plot on the selected descriptors for predicting the PIM1 inhibitory activity the majority of compounds from the data set are in this area, except one (compound **4**) from training set exceeds the threshold and it is considered as an outlier compound. This erroneous prediction could probably be attributed to the presence of sulfur on the R_1_ substituent whereas; the majority of compounds have an NH at this position.

While for the developed Williams plot on the selected descriptors for predicting the PIM2 inhibitory activity the majority of compounds from the data set are fallen within the AD, except two molecules: (compound **2**) in training set exceeds the threshold, so, it is considered as an outlier compound. Here, this erroneous prediction could probably be attributed to the unsubstituted R_2_ whereas; the majority of compounds are substituted at this position.

### Y-randomization

The Y-randomization method was carried out to validate the MLR models. Several random shuffles of the dependent variable (pIC_50_) were performed then after every shuffle, a QSAR was developed and the obtained results are shown in Table 4. The low Q^2^ and R^2^ values obtained after every shuffle indicate that the good result in our original MLR models are not due to a chance correlation of the training set.

**Table 4:** Q^2^ and R^2^ values after several Y-randomization tests

Iteration	MLR (PIM1)		MLR(PIM2)	
	Q^2^	R^2^	Q^2^	R^2^
1	−0.12	0.32	−0.48	0.82
2	−0.32	0.09	−0.09	0.03
3	−0.60	0.09	−0.14	0.33
4	−0.27	0.24	0.26	0.24
5	−0.19	0.12	0.31	0.07
6	−0.53	0.21	0.43	0.09
7	−0.34	0.11	0.45	0.19
8	−1.59	0.12	0.48	0.20
9	−0.77	0.09	0.54	0.23
10	−0.19	0.20	0.17	0.29

### External validation

To test the prediction ability of the obtained MLR models, it is required the use of a test set for external validation. As long as, the models generated on the training set using 19 of 5-(1H-indol-5-yl)-1,3,4-thiadiazol-2-amine derivatives were used to predict the PIM1 and PIM2 inhibitory activities of the remaining molecules. The parameters of the performance of the generated models are shown in Table 5. It can be seen clearly that the generated models are stable and predictable statically.

**Table 5:** The statistical results of MLR models with validation techniques

Method/parameter	R	R^2^	Q^2^	R^2^ test	MSE
MLR(PIM1)	0.87	0.726	0.60	0.84	0.222
MLR(PIM2)	0.91	0.825	0.73	0.74	0.184

Both obtained models for predicting the PIM1 and PIM2 inhibitory activities have high coefficients of determination for training (R^2^ = 0.726 and 0.825) and testing sets (test R^2^ = 0.84 and 0.74) respectively. Also high Cross-validation coefficients (Q^2^ = 0.60 and 0.76). So the proposed QSAR models can be used as primary step for screening and designing newly PIM1 and PIM2 inhibitors derived from 5-(1H-indol-5-yl)-1,3,4-thiadiazol.

### Screening of 5-(1H-indol-5-yl)-1,3,4-thiadiazol-2-amines analogues and prediction of their PIM1 and PIM2 inhibitory activities

**Fig. 5:**
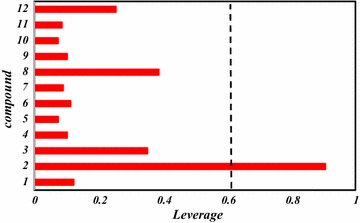
Leverage values of the screened compounds from pubchem database for the PIM1 inhibitory activity, listed in Table 7 (*h* ^*^ = 0.63)

**Fig. 6:**
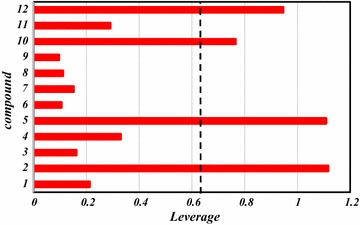
Leverage values of the screened compounds from the pubchem database for the PIM2 inhibitory activity, listed in Table 7 (*h* * = 0.63)

Overall, this study can be utilized to screen databases to look for new PIM1 and PIM2 inhibitors as well as to predict their inhibitory activities. Therefore, the built models were used to screen the Pubchem database, by searching compounds had 80% similarity with the most active compound of the studied series (compound **16**). Twelve compound were gathered as shown in Table 6 and their predicted values were calculated in addition to their leverages (*h*) to check if they fall in the AD of the proposed models (Table 6; Figs. 5, 6).

**Table 6:** Predicted values and calculated *h* of pIC_50_ (µM) according to different methods

Compound	Molecular structure	Pubchem CID	Pred (PIC_50_) for PIM1	*h*	Pred (PIC_50_) for PIM2	*h*
1		68328588	7.782	0.122	7.455	0.213
2		45377352	10.367	0.906	9.497	1.117
3		68328129	7.316	0.352	7.243	0.162
4		68327929	8.332	0.102	7.513	0.330
5		68328158	8.311	0.074	7.072	1.109
6		68328259	8.434	0.113	7.958	0.106
7		68328426	8.347	0.089	8.026	0.151
8		68328539	8.137	0.387	6.699	0.111
9		68328547	8.282	0.1023	7.594	0.0964
10		68328676	8.138	0.0739	7.068	0.7669
11		68328891	8.329	0.0860	8.244	0.2909
12		68356801	8.745	0.2543	8.137	0.9468

For the proposed model to predict the PIM1 inhibitory activity, almost of the compounds have *h* < *h* *, so their predicted values are regarded reliable except for compound **45377352** which has a leverage exceeds the threshold (*h* = 0.90).

While for the proposed model to predict the PIM2 inhibitory activity, it is found that among the twelve chemicals, only four were found to have *h* > *h* *, **45377352**, **68328158**, **68328676** and **68356801** respectively, so, expect for those molecules, the PIM2 predicted inhibitory activity of the eight remaining 5-(1H-indol-5-yl)-1,3,4-thiadiazol analogues is regarded reliable.

Moreover, the 5-(1H-indol-5-yl)-1,3,4-thiadiazol analogues were analyzed for their various properties, Log P, H-bond acceptor (H–A), H-bond donor (H–D), Polar surface area (P.S) (A^2^), Rotatable Bonds (R.B) and Molecular weight (MW) (g/mol), results shown that they follow the Lipinski’s rule of five for oral bioavailability [25]. Therefore, there are regarded to be acceptable as lead molecules to inhibit the PIM1 and PIM2 kinases.

## Conclusions

To predict the PIM1 and PIM2 inhibitory activities of a series substituted 5-(1H-indol-5-yl)-1,3,4-thiadiazol-2-amines, linear technique was used to propose useful mathematical models to establish quantitative relationships between them and a set of topological descriptors. Both proposed linear models MLR exhibit high determination coefficients, good stabilities and prediction abilities, using only three descriptors for each model. Such as the accuracy and predictability of the proposed models were checked based on the domain of applicability (AD), the Y-randomization and by comparing key statistical indicators, such as the R or R^2^ of the obtained models, as shown in Table 7. To validate these results, a test set was used, as shown in Table 5.

**Table 7:** Observed values and calculated values of pIC_50_ according to different methods

No	pIC_50_ (obs)	pIC_50_ PIM1 (pred)	pIC_50_ PIM2 (pred)
		MLR	MLR
1^a^	5.567	6.781	6.161
2^b^	5.996	6.769	5.581
3	5.787	6.744	4.604
4	4.657	5.440	6.154
5^a^	6.458	6.495	7.250
6^b^	6.010	6.708	7.455
7	6.983	7.793	6.373
8^a^	7.229	7.967	7.388
9^a, b^	7.431	7.791	7.708
10	7.677	7.781	7.446
11^b^	6.644	7.996	8.308
12	6.717	6.622	7.265
13	7.376	7.870	7.252
14	7.920	8.049	7.379
15	8.000	7.844	7.016
16	8.221	8.398	7.487
17	7.744	8.148	7.039
18	7.657	7.638	7.186
19	8.000	8.000	7.334
20	7.387	7.756	6.475
21	6.943	7.965	7.206
22^b^	7.552	7.689	8.003
23^a, b^	7.769	8.524	8.223
24^a^	7.602	8.408	5.947
25	5.889	7.578	7.268

^a, b^Are the test sets for PIM1 and PIM2 inhibitory activities respectively

Finally, we concluded that the topological descriptors used are able to encode the structural features of the studied compounds. Obviously, the obtained results from each model on this series of compounds were used as primary step for predicting the PIM1 and PIM2 inhibitory activity of 5-(1H-indol-5-yl)-1,3,4-thiadiazol analogues.

## Abbreviations

QSAR: quantitative structure activity relationship
PIM: proviral integration site for moloney murine leukaemia virus kinases
MLR: multiple linear regression
AD: applicability domain
GFA: genetic function algorithm
Q^2^: cross-validated determination coefficient
N: optimum number of components obtained from cross-validated PLS analysis and same used in final non-cross-validated analysis
R^2^: non-cross-validated correlation coefficient
MSE: standard error of the estimate
F: F test value
text R^2^: external validation determination coefficient
